# The Application of Diffusion Kurtosis Imaging on the Heterogeneous White Matter in Relapsing-Remitting Multiple Sclerosis

**DOI:** 10.3389/fnins.2022.849425

**Published:** 2022-03-10

**Authors:** Qiyuan Zhu, Qiao Zheng, Dan Luo, Yuling Peng, Zichun Yan, Xiaohua Wang, Xiaoya Chen, Yongmei Li

**Affiliations:** Department of Radiology, The First Affiliated Hospital of Chongqing Medical University, Chongqing, China

**Keywords:** multiple scleorsis (MS), magnetic resonance imaging (MRI), diffusion kurtosis imaging (DKI), normal-appearing white matter (NAWM), white matter lesions (WMLs), disease severity, cognitive impairment

## Abstract

**Objectives:**

To evaluate the microstructural damage in the heterogeneity of different white matter areas in relapsing-remitting multiple sclerosis (RRMS) patients by using diffusion kurtosis imaging (DKI) and its correlation with clinical and cognitive status.

**Materials and Methods:**

Kurtosis fractional anisotropy (KFA), fractional anisotropy (FA), mean kurtosis (MK), and mean diffusivity (MD) in T1-hypointense lesions (T1Ls), pure T2-hyperintense lesions (pure-T2Ls), normal-appearing white matter (NAWM), and white matter in healthy controls (WM in HCs) were measured in 48 RRMS patients and 26 sex- and age-matched HCs. All the participants were assessed with the Mini-Mental State Examination (MMSE), the Montreal Cognitive Assessment (MoCA), and the Symbol Digit Modalities Test (SDMT) scores as the cognitive status. The Kurtzke Expanded Disability Status Scale (EDSS) scores were used to evaluate the clinical status in RRMS patients.

**Results:**

The lowest KFA, FA, and MK values and the highest MD values were found in T1Ls, followed by pure-T2Ls, NAWM, and WM in HCs. The T1Ls and pure-T2Ls were significantly different in FA (*p* = 0.002) and MK (*p* = 0.013), while the NAWM and WM in HCs were significantly different in KFA, FA, and MK (*p* < 0.001; *p* < 0.001; *p* = 0.001). The KFA, FA, MK, and MD values in NAWM (*r* = 0.360, *p* = 0.014; *r* = 0.415, *p* = 0.004; *r* = 0.369, *p* = 0.012; *r* = −0.531, *p* < 0.001) were correlated with the MMSE scores and the FA, MK, and MD values in NAWM (*r* = 0.423, *p* = 0.003; *r* = 0.427, *p* = 0.003; *r* = −0.359, *p* = 0.014) were correlated with the SDMT scores.

**Conclusion:**

Applying DKI to the imaging-based white matter classification has the potential to reflect the white matter damage and is correlated with cognitive impairment.

## Introduction

Multiple sclerosis (MS) is one of the most prevalent chronic inflammatory demyelinated diseases, which is characterized by edema, inflammation, demyelination, and axonal loss in the central nervous system (Evangelou et al., 2000; Reich et al., 2018). To better diagnose and evaluate MS in clinical practice, magnetic resonance imaging (MRI), as a highly sensitive tool, can provide disease information and monitor the disease progression (Polman et al., 2011). White matter lesions (WMLs) can be detected as areas of hyperintensity on T2-weighted images (T2WI) or fluid-attenuated inversion recovery (FLAIR) images. Those T2-hyperintense lesions (T2Ls) correspond to a broad spectrum of pathological changes, including edema, inflammation, demyelination, remyelination, gliosis, liquid necrosis, and axonal loss (MacKay et al., 2006). Compared to T2Ls, however, non-enhancing T1-hypointense lesions (T1Ls) indicate more severe damage reflecting demyelination, axonal loss, gliosis, and loss of intracellular matrix and are thought to provide a more accurate classification of the microstructural damage in MS patients (Barkhof et al., 2009; Wolinsky et al., 2013). Therefore, we hypothesize that those differences in intensities of WMLs on T1-weighted images (T1WI) and T2WI/FLAIR represent the different pathological changes in the white matter, which contribute differently to the progression of the disease.

The conventional MRI can be used to evaluate the number, volume, and distribution of lesions in MS patients but lack the specific information of microstructural damage (Filippi et al., 2018). Diffusion tensor imaging (DTI) is widely used to evaluate the microstructural changes in the WMLs and normal-appearing white matter (NAWM) either in clinical practice or research, which provides more sensitive measures for clinically related brain abnormalities. However, the diffusion of water molecules in brain tissue often follows non-Gaussian distribution, which is not entirely characterized by DTI (Tuch et al., 2003). To better evaluate such microstructure, diffusion kurtosis imaging (DKI) has been proposed as an extension of DTI, which can quantify the non-Gaussian diffusion characteristics of water molecules in tissues, and provides more real and accurate tissue microstructure information than DTI (Jensen et al., 2005). DKI provides both diffusivity and kurtosis parameters. The diffusivity parameters, such as fractional anisotropy (FA) and mean diffusivity (MD), are used to assess white matter integrity, and the kurtosis parameters mainly include kurtosis fractional anisotropy (KFA) and mean kurtosis (MK). The KFA value summarizes the directional change of kurtosis, reflecting the anisotropy of kurtosis that can be used to describe a more complex environment of the brain tissue (Glenn et al., 2015). The MK value can reflect the microstructural complexity and the density of the axons and myelin, which has shown the better potential value to detect the tissue microstructure alterations in neurological disorders (Steven et al., 2014). The decreased MK values were found in the chronic demyelinated lesions, which is believed to be linked to a loss of microstructure (Falangola et al., 2014) while the increased MK values were found in the early acute inflammatory demyelinating phase of the lesions, which was associated with microgliosis (Guglielmetti et al., 2016). Thus, DKI sequence has the potential to reflect the microstructural damages or pathological changes in the white matter tissues (whether WMLs or NAWM).

Previous studies have paid much attention on the heterogeneity of MS lesions and made the classifications based on imaging (Thaler et al., 2015; Lipp et al., 2019; Ye et al., 2020). Some researchers have applied the advanced MRI techniques, including diffusion MRI (dMRI), to evaluate the heterogeneity of MS lesions or tissue states of white matter (Thaler et al., 2021). Furthermore, seeking for the correlation between disease severity and imaging metrics would make the classification of lesions or white matter tissues meaningful. When combining the dMRI-derived parameters with clinical or cognitive status, researchers applied DTI and DKI based on the different brain regions analysis and found the correlation between the white matter integrity and the cognitive status (de Kouchkovsky et al., 2016; Margoni et al., 2019; Schiavi et al., 2021). Despite all that, few studies evaluated the correlation between the different heterogeneous white matter and clinical or cognitive status based on the severity of the microstructural damage of white matter. We therefore hypothesize that the sensitive DKI-derived parameters would reveal some correlations with the disease severity when evaluating the different types of white matter tissue, which could be served for clinical practice.

Hence, the aims of this study were to (i) apply DKI to quantitatively evaluate the degree of microstructural damage in different white matter tissue types in a cohort of relapsing-remitting multiple sclerosis (RRMS) patients and healthy controls (HCs) and (ii) combine the results of the DKI-derived parameters with the clinical and cognitive status to explore the potential imaging biomarker and (iii) its correlation with severity of the disease.

## Materials and Methods

### Standard Protocol Approvals, Registrations, and Patient Consents

This retrospective study was approved by the Institutional Review Board of the First Affiliated Hospital of Chongqing Medical University, Chongqing, China, and written informed consent was obtained from each participant before MRI scans.

### Subjects

Forty-eight RRMS patients and 26 sex- and age-matched HCs were enrolled from the Department of Radiology, the First Affiliated Hospital of Chongqing Medical University. All the participants were recruited between July 2019 and December 2020. Inclusion criteria for MS patients were as follows: (1) a confirmed diagnosis of RRMS according to the 2017 revised McDonald’s diagnostic criteria (Thompson et al., 2018), (2) age 18–60 years, and (3) absence of neurological conditions other than MS. The exclusion criteria were as follows: (1) patients with contraindications for MRI scans, (2) history of intravenous corticosteroid treatment within 2 months before the imaging examinations, (3) image artifacts or incomplete clinical information, and (4) contrast-enhanced T1-MPRAGE hyperintense lesions. Among the 48 RRMS patients, only 10 of them were on the disease-modifying therapy (9 with teriflunomide and 1 with rituximab). The Mini-Mental State Examination (MMSE), the Montreal Cognitive Assessment (MoCA), and the Symbol Digit Modalities Test (SDMT) scores were used to assess the cognitive performance of all participants. The Kurtzke Expanded Disability Status Scale (EDSS) scores were used to assess the clinical status of the RRMS patients.

### Magnetic Resonance Imaging Data Acquisitions

All MR scans were performed on a 3-T MR scanner (Magnetom Skyra, Siemens Healthcare GmbH, Erlangen, Germany) using a 32-channel head coil. A standard protocol for MS studies included a sagittal 3-dimensional T1-weighted magnetization prepared rapid gradient echo (MPRAGE) sequence [echo-time (TE) = 2.26 ms, repetition time (TR) = 2,300 ms, inversion time (TI) = 900 ms, 192 slices, field of view (FOV) = 256 mm, voxel size = 1.0 × 1.0 × 1.0 mm, acquisition time (TA) = 5:21 min] and a sagittal 3-dimensional FLAIR (TE = 388 ms, TR = 5,000 ms, TI = 1,800 ms, 192 slices, FOV = 256 mm, voxel size = 0.5 × 0.5 × 1.0 mm, TA = 7:07 min). DKI data were acquired using the following parameters: TE = 97 ms, TR = 5,000 ms, 25 slices, FOV = 220 mm, voxel size = 1.7 × 1.7 × 4.0 mm, TA = 6:04 min, integrated Parallel Acquisition Techniques (iPAT) acceleration factor = 2 (GRAPPA), Partial-Fourier = 6/8, and three *b* values (0, 1,000, and 2,000 s/mm^2^) with diffusion encoding in 30 directions.

### Imaging Analysis

**FIGURE 1:**
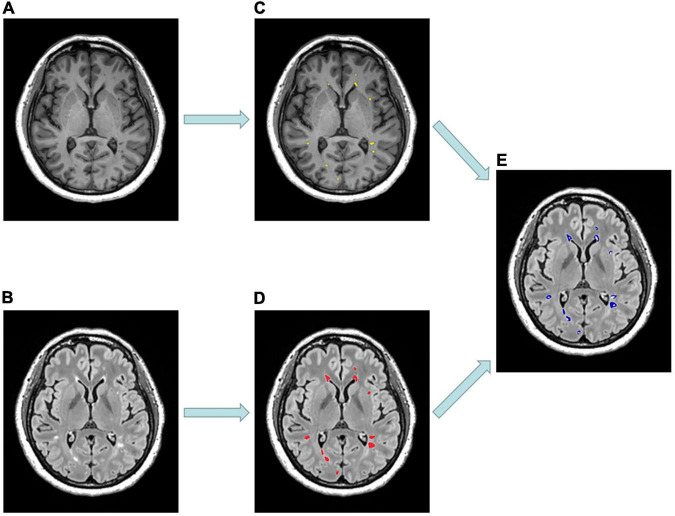
Example of MR scans and lesion masks of a 53-year-old patient diagnosed with relapsing-remitting multiple sclerosis. **(A)** Selected axial MPRAGE image with hypointense lesions. **(B)** Selected axial FLAIR image with hyperintense lesions. **(C)** T1-hypointense lesions (T1Ls) mask (yellow). **(D)** T2-hyperintense lesions (T2Ls) mask (red). **(E)** Pure T2-hyperintense lesions (pure-T2Ls) mask (blue) was obtained by subtracting **(C)** from **(D)**.

**FIGURE 2:**
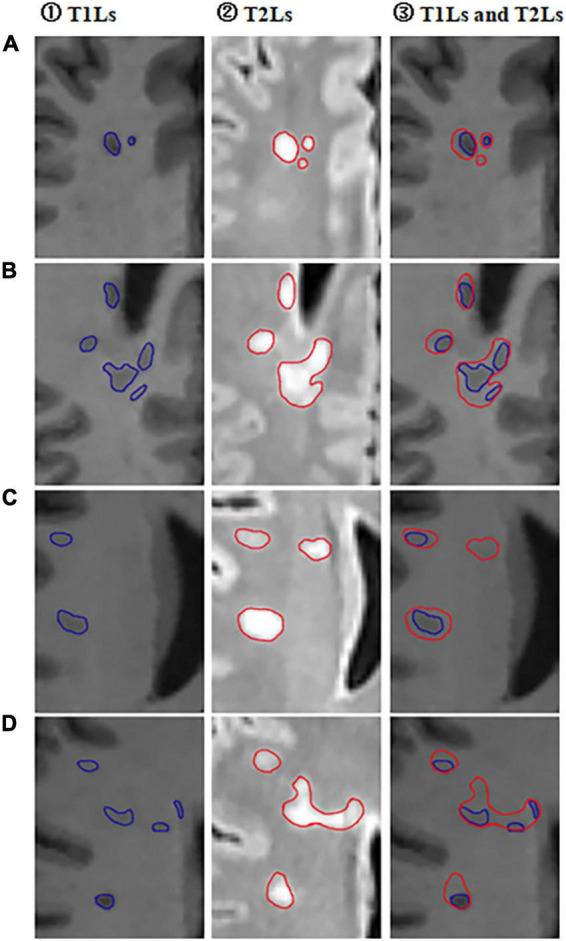
Selected partial axial images of MPRAGE and FLAIR sequences. **(A–D)** Partial axial FLAIR/MPRAGE images of four different RRMS patients in our cohort. ① Outlined T1-hypointense lesions (T1Ls) on MPRAGE images; ② Outlined T2-hyperintese (T2Ls) lesions on FLAIR images; ③ Outlined both T2-hyperintense lesions (T1Ls) and T1-hypointense lesions (T2Ls) on MPRAGE images.

In each RRMS patient, we purposely divided the white matter into three different areas, including T1-weighted hypointense lesions with corresponding T2-weighted hyperintensity defined as T1Ls, T2-weighted/FLAIR hyperintense lesions without T1-weighted hypointensity defined as pure-T2Ls, and normal-appearing white matter defined as NAWM (Figures 1, 2). The total T2-hyperintense lesions volume (T2LV), T1Ls volume (T1LV), and pure-T2Ls volume (pure-T2LV) were calculated in each patient.

T1Ls were defined as areas with hypointense on T1-weighted images and hyperintense on a T2-weighted/FLAIR image. Thus, two independent raters (XC and DL) manually outlined the hypointense lesions according to the definition above on T1-weighted MPRAGE images using the ITK-SNAP 3.8.0 (accessed on June of 2019)^1^ (Yushkevich et al., 2006). The resulting T1L masks of both raters were binarized and patient-wise multiplied to obtain a consensus mask.

As mentioned above, pure-T2Ls were defined as areas with only hyperintense on T2-weighted/FLAIR images and without hypointensity on T1-weighted images. The FLAIR image of each RRMS patient was registered to the T1-MPRAGE image. All the T2-hyperintense lesions were semi-automatically segmented on FLAIR images. Both the two steps mentioned above were using the Lesion Segmentation Tool (Schmidt et al., 2012). For accuracy, the semi-automatically segmented T2-hyperintense lesion masks were manually controlled and corrected to obtain the T2L masks. Since all T1Ls were shown as T2 hyperintense lesions in each patient, the T1L mask was subtracted from the T2L mask to obtain only the pure-T2L mask.

For the NAWM mask, we subtracted the T2L mask from the whole brain white matter mask, leaving those areas that are isointense both on T1-weighted and FLAIR/T2-weighted images. The whole-brain white matter mask was obtained from T1-weighted MPRAGE imaging by using Voxel-based morphometry (VBM) (Ashburner and Friston, 2000) in SPM8 software.^2^

Additionally, in the HCs cohort, we extracted the whole brain white matter (WM) mask in each subject. All the image binary subtraction steps mentioned above used the MATLAB R2013b software.

### Diffusion Kurtosis Imaging Processing

**FIGURE 3:**
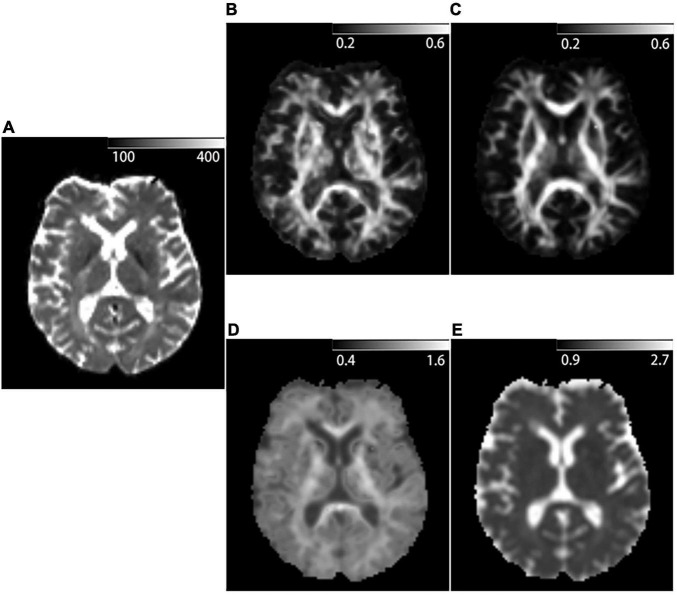
Example of DKI-derived parametric maps of a 53-year-old patient diagnosed with Relapsing-remitting multiple sclerosis. **(A)** *b* = 0 mm^2^/s image acquired with DKI sequence. **(B)** Kurtosis fractional anisotropy (KFA) parametric map acquired with DKI sequence. **(C)** Fractional anisotropy (FA) parametric map acquired with DKI sequence. **(D)** Mean kurtosis (MK) parametric map acquired with DKI sequence. **(E)** Mean diffusivity (MD) parametric map acquired with DKI sequence.

DKI data were pre-processed, including denoising (Veraart et al., 2016), removal of Gibbs ringing artifacts (Kellner et al., 2016), correction of subject motion (Leemans and Jones, 2009) using the MRtrix3 package (Tournier et al., 2019), eddy-currents (Andersson et al., 2017), and susceptibility-induced distortions (Andersson et al., 2003) in FMRIB Software Library (FSL)^3^ (Jenkinson et al., 2012). All the T1 images were registered to DKI b0 space individually using FSL. The diffusion kurtosis parametric maps, including the kurtosis fractional anisotropy (KFA), fractional anisotropy (FA), mean kurtosis (MK), and mean diffusivity (MD) (Figure 3), were calculated by the diffusion kurtosis estimator software (DKE),^4^ using constrained linear least-squares quadratic programming (CLLS-QP) algorithm and applying standard parameters (spatial smoothing and strong median filtering) (Tabesh et al., 2011). The details of parameters used with DKE were as follows: the full width at half maximum (FWHM) of the Gaussian kernel was 3.375 mm; *Kmin* = 0, *NKmax* = 3 and *D* > 0 for constraint on directional kurtosis; 0 < *K* < 3 for thresholds on output kurtosis maps. DKI tensor fitting was performed with constrained linear weighted fitting and DTI tensor fitting was performed with linear weighted fitting to, respectively, obtain the DKI- and DTI-based parametric maps. Finally, the binarized masks were multiplied with the corresponding KFA, FA, MK, and MD maps to obtain mean KFA, FA, MK, and MD values for each white matter tissue type of masks.

### Statistical Analysis

Statistical analysis was performed using SPSS version 26.0 (IBM Corp., Armonk, NY, United States) and GraphPad Prism V5. Kolmogorov–Smirnov tests were applied to assess the normality of data distribution. Between RRMS patients and HCs, independent samples *t*-tests were used for age, MMSE, MoCA, and SDMT scores analysis and the Chi-square test was used for gender analysis. One-way ANOVA with the Bonferroni correction for multiple comparisons was used to compare the DKI parameter values in the four types of different white matter areas (T1Ls, pure-T2Ls, NAWM, and WM in HCs). *p*-values < 0.05 were considered statistically significant. Partial correlation analysis using age and education years as covariates was used to evaluate the relationship between T1LV, T2LV, pure-T2LV, and DKI parameter values with EDSS scores and the neuropsychological scores (MMSE, MoCA, and SDMT scores). To make the EDSS scores and the neuropsychological scores comparable with the imaging parameters, the *z*-scores of the four scores were calculated for each RRMS patient before the partial correlation analysis.

## Results

### Demographic and Clinical Data

Demographic data and clinical characteristics of the participants are shown in Table 1. There were no significant differences in gender (*p* = 0.945) and age (*p* = 0.075) between RRMS patients and HCs. There were significant differences in MMSE (*p* = 0.013), MoCA (*p* = 0.016), and SDMT (*p* < 0.001) between RRMS patients and HCs.

**TABLE 1:** Demographic and clinical characteristics of the participants.

	RRMS patients	Healthy controls	*p*
No. of participants	48	26	
Age (years)	33.1 ± 9.2	37.0 ± 8.3	0.075*^a^*
Sex (male/female)	17/31	9/17	0.945*^b^*
DD (years)	5.4 ± 5.2	-	-
Education (years)	14.1 ± 2.9	14.3 ± 4.5	0.85*^a^*
EDSS scores	2.2 ± 1.3	-	-
MMSE scores	28.6 ± 1.6	29.3 ± 0.8	0.013*^a^*
MoCA scores	25.5 ± 3.5	27.4 ± 2.4	0.016*^a^*
SDMT scores	42.6 ± 17.3	53.2 ± 9.7	0.001*^a^*
T1LV (mm^3^)	14,615.5 ± 11,939.4	-	-
T2LV (mm^3^)	22,998.9 ± 19,421.2		-
Pure-T2LV (mm^3^)	8,383.4 ± 8,571.3		-

*^a^p obtained using independent samples t-tests.*

*^b^p obtained using the Chi-square test.*

*The data were shown as the mean values ± standard deviation. DD, disease duration; EDSS, Expanded Disability Status Scale; MMSE, Mini-Mental State Examination; MoCA, Montreal Cognitive Assessment; SDMT, Symbol Digit Modalities Test.*

### Diffusion Kurtosis Imaging Parameter Analysis

**FIGURE 4:**
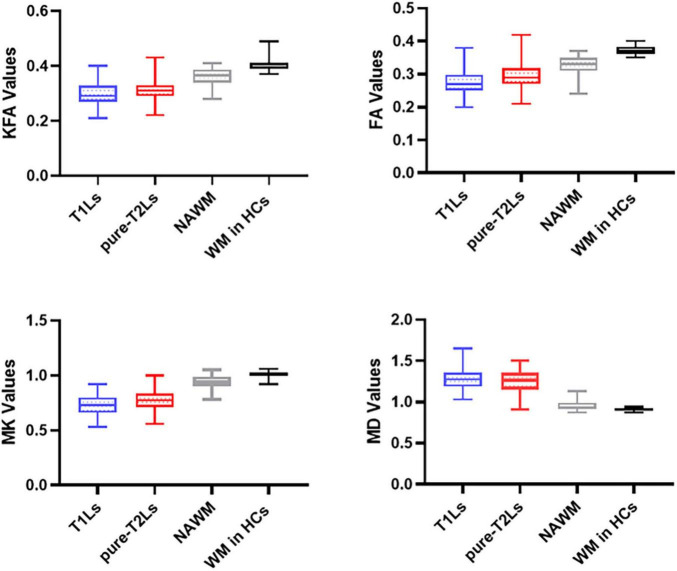
Distribution of DKI parameters values in different white matter tissue types. T1Ls, T1-hypointense lesions; pure-T2Ls, pure T2-hyperintense lesions; NAWM, normal-appearing white matter; KFA, kurtosis fractional anisotropy; FA, fractional anisotropy; MK, mean kurtosis; MD, mean diffusivity.

The distribution of DKI parameter values in four tissue types is displayed in Figure 4. The four DKI parameter values were significantly different among the four tissue types, which are reported in Table 2. The *post hoc* analysis results are reported in Table 3. The lowest FA and MK values were shown in T1Ls, followed by pure-T2Ls, NAWM, and WM in HCs, and they were significant between each type of tissue (*p* < 0.05). Like the results of MK values, the lowest KFA and highest MD values were obtained in the T1Ls, followed by pure-T2Ls, NAWM, and WM in HCs. Except between T1Ls and pure-T2Ls in KFA (*p* = 0.388) and MD (*p* = 1.000) and between NAWM and WM in HCs in MD (*p* = 0.671), significant differences of KFA and MD values were found between every other two white matter tissue types (*p* < 0.001).

**TABLE 2:** The DKI parameter values analysis within the four white matter tissue types.

	KFA	FA	MK	MD
T1Ls (48)	0.295 ± 0.040	0.271 ± 0.039	0.727 ± 0.093	1.286 ± 0.142
Pure-T2Ls (48)	0.308 ± 0.036	0.295 ± 0.039	0.776 ± 0.090	1.256 ± 0.132
NAWM (48)	0.361 ± 0.030	0.327 ± 0.029	0.938 ± 0.064	0.949 ± 0.057
WM in HCs (26)	0.408 ± 0.027	0.372 ± 0.014	1.009 ± 0.029	0.907 ± 0.017
*F*	78.161	58.459	111.594	137.334
*p*	<0.001	<0.001	<0.001	<0.001

*T1Ls, T1-hypointense lesions; pure-T2Ls, pure T2-hyperintense lesions; NAWM, normal-appearing white matter; WM in HCs, white matter in healthy controls; KFA, kurtosis fractional anisotropy; FA, fractional anisotropy; MK, mean kurtosis; MD, mean diffusivity.*

**TABLE 3:** *Post hoc* analysis between the DKI parameter values among the four white matter tissue types.

	KFA		FA		MK		MD	
	*t*	*p*	*t*	*p*	*t*	*p*	*t*	*p*
T1Ls vs. pure-T2Ls	–1.861	0.388	–3.620	0.002**	–3.112	0.013*	1.350	1
T1Ls vs. NAWM	–9.307	< 0.001***	–8.194	< 0.001***	–13.345	< 0.001***	15.306	< 0.001***
T1Ls vs. WM in HCs	–13.384	< 0.001***	–12.386	< 0.001***	–14.946	< 0.001***	14.432	< 0.001***
Pure-T2Ls vs. NAWM	–7.448	< 0.001***	–4.575	< 0.001***	–10.232	< 0.001***	13.955	< 0.001***
Pure-T2Ls vs. WM in HCs	–11.825	< 0.001***	–9.352	< 0.001***	–12.338	< 0.001***	13.300	< 0.001***
NAWM vs. WM in HCs	–5.582	< 0.001***	–5.517	< 0.001***	–3.763	< 0.001***	1.599	0.671

Statistical differences are *p < 0.05, **p < 0.01, ***p < 0.001.

*T1Ls, T1-hypointense lesions; pure-T2Ls, pure T2-hyperintense lesions; NAWM, normal-appearing white matter; WM in HCs, white matter in healthy controls; KFA, kurtosis fractional anisotropy; FA, fractional anisotropy; MK, mean kurtosis; MD, mean diffusivity.*

### Correlations Between Imaging Parameters and Neuropsychological Scores

The MK values of pure-T2Ls (*r* = 0.331, *p* = 0.024) and the KFA, FA, MK, and MD values of NAWM (*r* = 0.360, *p* = 0.014; *r* = 0.415, *p* = 0.004; *r* = 0.369, *p* = 0.012; *r* = −0.531, *p* < 0.001) were correlated with the MMSE scores. The FA values of pure-T2Ls (*r* = 0.309, *p* = 0.036) were correlated with the MoCA scores. The FA and MK values of T1Ls (*r* = 0.322, *p* = 0.029; *r* = 0.371, *p* = 0.011), the FA and MK values of pure-T2Ls (*r* = 0.355, *p* = 0.015; *r* = 0.400, *p* = 0.006), and the FA, MK, and MD values of NAWM (*r* = 0.423, *p* = 0.003; *r* = 0.427, *p* = 0.003; *r* = −0.359, *p* = 0.014) were correlated with the SDMT scores. The T1LV (*r* = −0.409, *p* = 0.005) and T2LV (*r* = −0.360, *p* = 0.014) were correlated with the SDMT scores. There was no correlation between the DKI-derived parameters and the EDSS scores (Table 4).

**TABLE 4:** Correlation between imaging parameters and clinical or cognitive assessment.

	EDSS(z)		MMSE(z)		MoCA(z)		SDMT(z)	
	*r*	*p*	*r*	*p*	*r*	*p*	*r*	*p*
T1LV	0.166	0.270	–0.178	0.238	–0.013	0.931	–0.360	0.014*
T2LV	0.185	0.21	–0.174	0.247	0.012	0.939	–0.409	0.005**
Pure-T2LV	0.119	0.433	–0.159	0.290	–0.046	0.761	–0.246	0.099
T1Ls_KFA	–0.115	0.445	0.056	0.712	0.093	0.538	0.000	1.000
T1Ls_FA	–0.167	0.266	0.148	0.325	0.076	0.617	0.322	0.029*
T1Ls_MK	–0.197	0.190	0.270	0.069	–0.070	0.643	0.371	0.011*
T1Ls_MD	–0.275	0.065	–0.237	0.113	0.124	0.411	–0.199	0.185
Pure-T2Ls_KFA	–0.062	0.680	0.097	0.522	0.278	0.061	0.740	0.627
Pure-T2Ls_FA	–0.112	0.458	0.218	0.146	0.309	0.036*	0.355	0.015*
Pure-T2Ls_MK	–0.099	0.513	0.331	0.024 < *cps*:*it* > * < /*cps*:*it* >	0.142	0.345	0.400	0.006**
Pure-T2Ls_MD	–0.150	0.321	–0.255	0.087	–0.086	0.570	–0.146	0.331
NAWM_KFA	–0.142	0.347	0.360	0.014*	0.267	0.073	0.261	0.080
NAWM_FA	–0.259	0.082	0.415	0.004**	0.176	0.241	0.423	0.003**
NAWM_MK	–0.157	0.296	0.369	0.012*	0.079	0.602	0.427	0.003**
NAWM_MD	–0.198	0.188	–0.531	< 0.001***	–0.209	0.162	–0.359	0.014*

Statistical differences are *p < 0.05, **p < 0.01, ***p < 0.001.

*T1LV, T1-hypointense lesions volume; T2LV, T2-hyperintense lesions volume; pure-T2LV, pure T2-hypointense lesions volume; T1Ls, T1-hypointense lesions; pure-T2Ls, pure T2-hyperintense lesions; NAWM, normal-appearing white matter; KFA, kurtosis fractional anisotropy; FA, fractional anisotropy; MK, mean kurtosis; MD, mean diffusivity; EDSS, Expanded Disability Status Scale; MMSE, Mini-Mental State Examination; MoCA, Montreal Cognitive Assessment; SDMT, Symbol Digit Modalities Test.*

## Discussion

In this study, we applied DKI to characterize the microstructural damage in the heterogeneity of different white matter areas and examine the correlations between the various degrees of DKI-derived parameter changes in different white matter types and the clinical or cognitive status. We found the lowest KFA, FA, and MK values, respectively, in T1Ls, followed by pure-T2Ls, NAWM, and WM in HCs. The highest MD values were found in T1Ls on the contrary, followed by pure-T2Ls, NAWM, and WM in HCs. We observed that the MK could be well-distinguished T1Ls and pure-T2Ls, as well as NAWM and WM in HCs. The results above indicated that the most severe microstructural damage occurred in T1Ls, which presented a different pathological state from pure-T2Ls. At the same time, NAWM was significantly different from WM in HCs, even if it had minor histopathological damage compared to focal lesions. Nevertheless, the damage degree reflected by DKI parameters in WMLs (T1Ls and pure-T2Ls) had little correlation with the clinical disability or cognitive impairment. Instead, DKI parameters in the NAWM were correlated with the cognitive status.

Previous studies have largely focused on the heterogeneity of different lesion types or tissue types in the white matter by using advanced MRI techniques. Faizy et al. (2016) have observed that different types of MS lesions showed significant differences by measuring myelin water fraction (MWF) values, presumably related to differences in the degree of tissue destruction. Lipp et al. (2019) quantified white matter damage *in vivo* by measuring fractional anisotropy (FA), radial diffusivity (RD), MWF, and magnetization transfer ratio (MTR) to differentiate the white matter tissue states in MS patients. Schiavi et al. (2021) used DBSI and DTI sequences of a 7-T scanner to investigate the pathology of different white matter tissue. Our present study was similar in lesion or white matter classifications with the previous studies above. However, the application of DKI based on ROI analysis in the different lesion or white matter categories is still rare.

DKI can characterize the non-Gaussian distribution of water molecules (Wu and Cheung, 2010). The diffusion kurtosis can reflect the deviation of water molecules from Gaussian distribution (Hui et al., 2008). The higher the diffusion kurtosis, the more the water molecule diffusion deviates from Gaussian distribution, indicating a more restricted diffusion and complex environment. KFA, one of the DKI-derived parameters, is similar to FA to some extent. However, KFA can better describe complex diffusion profiles due to the lesser non-isotropic diffusion effect and the high *b*-values of the DKI model (Lee et al., 2018; Li et al., 2021). Furthermore, it can also provide an essential complement for the poor performance of FA when the packing density of fiber bundles and axons increases (Karlsen et al., 2019). Notwithstanding, most studies did not use KFA values to evaluate white matter microstructure changes. Here, we utilized KFA values to investigate the varying degrees of microstructural damage in white matter and found that KFA values were the lowest in the most severe areas (T1Ls), just as FA values. However, FKA values are less sensitive in distinguishing the four types of white matter tissues than FA values in our results. The difference in KFA values between T1Ls and pure-T2Ls did not reach significance. That may explain why only a few studies have used KFA values in detecting the white matter microstructure, even if KFA values can better describe the real biological tissue than FA values (Karlsen et al., 2019; Li et al., 2021). While the DKI-derived FA values in our study are in line with the previous studies (Yoshida et al., 2013; Lipp et al., 2019), the FA values were significantly different in each two types, respectively, between the four white matter tissue types.

Thaler et al. (2021) have emphasized the MK and MD values when applying DKI to the heterogeneity of MS lesions. Consistent with their findings, we found that the MK values offered a better discriminative effect than the MD values and might become a potential image biomarker in distinguishing the different lesion types. There was a significant difference in MK values between each two white matter tissue types, including between T1Ls and pure-T2Ls and between NAWM and WM in HCs. However, they did not observe a significant difference between NAWM and WM in HCs, which may be because they used a manually drawn ROIs-based approach to select eight regions of NAWM while we extracted the whole brain NAWM. Nevertheless, the MD values did not show good performance neither in distinguishing the two WMLs types nor in distinguishing NAWM and WM in HCs. Just as its name implies, MK values are the average kurtosis value in all directions, which can reflect the microstructure complexity and integrity (Grinberg et al., 2017). The decreased MK values indicated a shift of a diffusion kurtosis toward free water and compromised microstructure complexity (Coutu et al., 2014), which may be caused by axonal loss, myelin sheath damage, and the destruction of cellular components. Hence, the result of the lowest MK values in T1Ls showed the place where the most destructive white matter damage occurred. These findings demonstrated that DKI-derived parameters, especially the MK values, were closely linked to structural and intensity abnormalities seen on conventional MRI and were able to show abnormalities in NAWM that conventional MRI could not image for MS patients.

It is worth noting that most of the WMLs consist of T1Ls and pure-T2Ls in this work. T1Ls are in the center of these lesions, while pure-T2Ls are in the lesions’ border. As previously mentioned, all the DKI-derived parameters were more altered in T1Ls, whether significantly or not, which means that the severe tissue damage and axon loss occurred in the center of the lesions while the white matter structure was more complex at the lesion’s border. That relatively microstructure complexity in pure-T2Ls (WMLs’ border) might result from activated microglia and macrophages at the rim of chronic active lesions (Dal-Bianco et al., 2017). This assumption was in line with the previous studies (Gillen et al., 2018; Schiavi et al., 2021) and could better explain the fact that the white matter areas of pure-T2Ls were presented differently on the two different sequences.

Furthermore, we sought to determine whether the DKI parameter values in the most severe areas (T1Ls) were related to the clinical or cognitive status. We used EDSS scores to reflect the clinical disability and neuropsychological scores such as MMSE, MoCA, and SDMT scores to reflect the cognitive functions. Disappointingly, the DKI parameter values were barely correlated with the clinical or cognitive status neither in T1Ls nor pure-T2Ls. These results might be explained by the experimental design. In this cross-sectional study, we only selected the image data at a certain time to draw the ROIs of WMLs instead of grouping newly arising lesions or persistent lesions since these two kinds of lesions contribute to clinical disability and cognitive impairment differently (Masek et al., 2008; Louapre et al., 2017). In our RRMS cohort, newly arising lesions, considered to be more related to the progression in clinical disability and cognitive impairment (Summers et al., 2008; Rovira et al., 2013; Eijlers et al., 2018), may only account for an extremely small proportion of all the inclusive WMLs. The EDSS scores reflect several domains including vision, speech, sensorimotor, and bladder function, lacking specificity, which may cause the poor correlation with the imaging parameters. However, most DKI parameter values in NAWM were related to the cognitive decline, mainly manifested by MMSE and SDMT scores. Previous studies have applied quantitative MRI to detect the injury of NAWM when exploring the cognitive impairment in patients with MS. At the same time, our results are consistent with previous myelin water imaging (MTI) and DTI studies. MTI has proved the efficacy in evaluating the relationship between NAWM myelin heterogeneity and cognitive processing speed performance by calculating the MWF (Abel et al., 2020). The loss of NAWM integrity has demonstrated the correlation with the cognitive impairment in a cohort of pediatric MS patients by measuring the DTI parameters (Bethune et al., 2011). Since NAWM is diffusely abnormal in patients with MS (Elliott et al., 2021) and has a more pronounced correlation with clinical progression (Filippi et al., 2012), DKI could become an effective tool to not only characterize widespread microstructural damage in the NAWM but also reflect the severity of the disease by imaging.

Moreover, among the four DKI-derived parameters, only MK values in T1Ls, pure-T2Ls, and NAWM (*p* = 0.011, *r* = 0.371; *p* = 0.006, *r* = 0.400; *p* = 0.003, *r* = 0.427) were correlated with the SDMT scores. To our knowledge, although de Kouchkovsky et al. (2016) reported that DKI-based white matter tract integrity (WMTI) in NAWM significantly correlated with SDMT scores, and DKI-based axonal water fraction (AWF) in NAWM associated with EDSS scores, while Nygaard et al. (2021) found that the DKI-derived parameters in cortex were associated with walking and cognitive performance, this is the first study to demonstrate that the widespread white matter damage (including WMLs and NAWM) in RRMS patients reflected by MK values is correlated with the cognitive status reflected by SDMT scores. Combined with the good performance of MK values in distinguishing the four tissue types mentioned above, therefore, MK values could be a potential biomarker to evaluate the white matter damage and its correlation with the cognitive impairment.

There are several limitations in our study. First, as previously mentioned, this is a cross-sectional study, and we could not detect the newly arising lesions and calculate the increase of the accumulation of lesions volume longitudinally. Further studies will be needed to longitudinally compare the newly arising lesion microstructure changes with other white matter tissue types and investigate its relationship with the clinical progression. Second, we must admit the difficulty of obtaining pathological data. Since the pathological correlation was not available for our research group, we aimed to provide insights into clinico-radiological features avoiding pathology to evaluate the disease severity by the imaging parameters and find the potential biomarker of clinically relevant white matter injury. Last, in this work, we only used the DKI sequence to quantitatively evaluate the white matter microstructural damage. Other advanced MRI techniques such as T1 mapping, quantitative susceptibility mapping (QSM), ultra-short echo time (UTE), and MTR will be necessarily selected for further investigation.

## Conclusion

In conclusion, our findings demonstrated the heterogeneity of white matter microstructure changes, which could be well-distinguished by DKI parameters. The damage in NAWM reflected by DKI parameters correlated to the cognitive impairment, and the MK value could be a potential biomarker to evaluate the white matter damage and its correlation with the cognitive impairment. This imaging-based classification in white matter may help evaluate the disease severity and progression.

## Data Availability Statement

The raw data supporting the conclusions of this article will be made available by the authors, without undue reservation.

## Ethics Statement

The studies involving human participants were reviewed and approved by the Institutional Review Board of the First Affiliated Hospital of Chongqing Medical University, Chongqing, China. The patients/participants provided their written informed consent to participate in this study. Written informed consent was obtained from the individual(s) for the publication of any potentially identifiable images or data included in this article.

## Author Contributions

QZu and XC offered the research idea. QZu prepared the manuscript. XC and QZe offered the data processing support. XC and DL assisted in imaging processing. QZe assisted in completing the statistics and experimental design. QZe, XC, DL, YP, ZY, XW, and YL provided guidance and critical reviews. All authors contributed to the article and approved the submitted version.

## Conflict of Interest

The authors declare that the research was conducted in the absence of any commercial or financial relationships that could be construed as a potential conflict of interest.

## Publisher’s Note

All claims expressed in this article are solely those of the authors and do not necessarily represent those of their affiliated organizations, or those of the publisher, the editors and the reviewers. Any product that may be evaluated in this article, or claim that may be made by its manufacturer, is not guaranteed or endorsed by the publisher.

## Footnotes

1
http://itksnap.org

2
https://www.fil.ion.ucl.ac.uk/spm/

3
https://fsl.fmrib.ox.ac.uk/fsl/fslwiki/

4
https://www.nitrc.org/projects/dke

## Abbreviations

RRMS: Relapse-remitting multiple sclerosis
HCs: Healthy controls
WMLs: White matter lesions
T1Ls: T1-hypointense lesions
T2Ls: T2-hyperintense lesions
NAWM: Normal-appearing white matter
WM in HCs: White matter in healthy controls
DKI: Diffusion kurtosis imaging
KFA: Kurtosis fractional anisotropy
FA: Fractional anisotropy
MK: Mean kurtosis
MD: Mean diffusivity
MMSE: Mini-Mental State Examination
MoCA: Montreal Cognitive Assessment
SDMT: Symbol Digit Modalities Test
EDSS: Expanded Disability Status Scale.
